# Efficacy and safety of intravenous golimumab plus methotrexate in patients with rheumatoid arthritis aged < 65 years and those ≥ 65 years of age

**DOI:** 10.1186/s13075-019-1968-x

**Published:** 2019-08-20

**Authors:** John Tesser, Shelly Kafka, Raphael J. DeHoratius, Stephen Xu, Elizabeth C. Hsia, Anthony Turkiewicz

**Affiliations:** 1 Arizona Arthritis and Rheumatology Associates, Phoenix, AZ USA; 2 Janssen Scientific Affairs, LLC, Horsham, PA USA; 3 Sidney Kimmel School of Medicine, Thomas Jefferson University, Philadelphia, PA USA; 4 Janssen Research & Development, LLC, Spring House, PA USA; 5 University of Pennsylvania, Philadelphia, PA USA; 6 Rheumatology Associates, Birmingham, AL USA

**Keywords:** Rheumatoid arthritis, Geriatric, Anti-tumor necrosis factor, Golimumab

## Abstract

**Objective:**

To evaluate the safety and efficacy of intravenous golimumab + methotrexate (MTX) in patients with active rheumatoid arthritis (RA) aged < 65 years and those ≥ 65 years who were enrolled in the GO-FURTHER study.

**Methods:**

In the phase III, double-blind, randomized, placebo-controlled GO-FURTHER trial, patients with active RA were randomized to intravenous (IV) golimumab 2 mg/kg + MTX or placebo + MTX at weeks 0 and 4, then every 8 weeks thereafter (with crossover to golimumab at week 16 [early escape] or week 24 [per-protocol]). The final golimumab infusion was at week 100. Assessments included American College of Rheumatology (ACR) 20/50/70 response criteria. Efficacy and adverse events (AEs) were monitored through 2 years. Efficacy and AEs were summarized for patients aged < 65 years or ≥ 65 years; AEs were also summarized for patients < or ≥ 70 years and patients < or ≥ 75 years.

**Results:**

In GO-FURTHER, 592 patients were randomized to receive placebo (*n* = 197) or golimumab (*n* = 395), 515 were aged < 65 years and 77 were ≥ 65 years. At week 24, ACR20 response rates were greater for golimumab + MTX patients compared with placebo + MTX for patients < 65 years (61.6% vs 31.3%, *p* < 0.001) and those ≥ 65 years (69.5% vs 33.3%; *p* < 0.01). Infections were the most common AE through week 112 (51.6% in patients < 65 years; 55.3% in patients ≥ 65 years); upper respiratory infections were the most common infection in patients < 65 years (13.2%) and those ≥ 65 years (11.8%). Serious AEs occurred in 17.7% in patients < 65 years and 25.0% of patients ≥ 65 years and included malignancies, pneumonia, fractures, acute pancreatitis, cellulitis, and bacterial arthritis.

**Conclusions:**

In GO-FURTHER, ACR response rates were similar between patients < 65 years and patients ≥ 65 years within each treatment group. AEs in elderly patients were similar to the known safety profile of IV golimumab. Immunosenescence is known to increase the risk of infections in the elderly. Elderly patients had a numerically higher incidence of serious infections. Six malignancies occurred in golimumab-treated patients, all in patients < 65 years.

**Trial registration:**

clinicaltrials.gov: NCT00973479. Registered September 9, 2009.

## Background

Biologics targeting tumor necrosis factor (TNF) are an effective therapy for reducing the inflammation and improving the signs and symptoms of rheumatoid arthritis (RA). Golimumab is a fully human monoclonal anti-TNF antibody and is available as a subcutaneous (SC) injection or an intravenous (IV) infusion. The safety profile of golimumab has been established from trials of SC golimumab in patients with RA [1–3], psoriatic arthritis (PsA) [4], ankylosing spondylitis (AS) [5], and ulcerative colitis [6] and from trials of IV golimumab in patients with RA [7], PsA [8], and AS [9]. The adverse events (AEs) reported in these trials have been consistent with those reported for other anti-TNF therapies. Overall, patients with RA who are treated with anti-TNF therapies are at an increased risk for infections and serious infections, including opportunistic infections, and screening for tuberculosis is recommended as described in the prescribing information [10–14]. In addition, other AEs of interest include malignancies and cardiovascular events.

In clinical trials and post-marketing experience in patients with RA, the proportions of patients who had a serious AE while receiving golimumab SC or IV have been low. However, there is a paucity of data of the safety of golimumab, and anti-TNF agents in general, among patients aged 65 years and older. In one study of patients with RA who were 65 years and older, there was no increase in the incidence of serious bacterial infections for patients receiving anti-TNF therapy when compared with patients treated with methotrexate (MTX) only; however, the use of glucocorticoids was associated with an increased risk [15]. In a retrospective analysis of patients with RA enrolled in a Korean registry, the rates of infections and malignancies were higher in patients 65 years and older than in patients who were younger, although these differences were not statistically significant [16]. In another analysis of data from four randomized trials of patients with RA treated with etanercept, the rates of serious adverse events (SAEs) and serious infections were higher in older patients compared with younger patients in three of the studies, although no difference was seen in the fourth study [17]. In the same analysis, the rate of malignancies among elderly patients was higher than for younger patients, but was consistent with the same age group in the general population [17].

In the phase 3 GO-FURTHER study, the safety and efficacy of IV golimumab 2 mg/kg was evaluated in adults with active RA despite treatment with MTX [18]. Safety events that occurred during GO-FURTHER were consistent with those previously reported with SC golimumab in patients with RA [1–3], PsA [4], and AS [5]. We performed an exploratory analysis to compare the safety and efficacy following golimumab plus MTX therapy in patients aged 65 years and older who were enrolled in the GO-FURTHER study.

## Methods

### Patients and study design

The details of the GO-FURTHER patient population and study design have been previously published [18]. The GO-FURTHER trial was a phase 3, randomized, placebo-controlled trial. Adults with active RA (≥ 6 swollen and ≥ 6 tender joints) for ≥ 3 months despite MTX therapy were eligible for enrollment. Patients also had to have a screening C-reactive protein level ≥ 1.0 mg/dL and a positive status for either anti-cyclic citrullinated peptide antibodies or rheumatoid factor. Patients who were receiving MTX had to have been receiving a stable dose (≥ 15 mg/week) for ≥ 3 months at screening and a stable dose (15–25 mg/week) for ≥ 4 weeks prior to enrollment. Concomitant use of oral corticosteroids (≤ 10 mg/day of prednisone or equivalent) and nonsteroidal anti-inflammatory drugs (NSAIDs) (or other analgesics for RA, at usual approved doses) was permitted at stable doses [18]. Prior biologic therapy was not permitted, but patients could have received prior treatment with disease-modifying anti-rheumatic drugs (DMARDs) (other than MTX) and systemic immunosuppressives (e.g., d-penicillamine, hydroxychloroquine, chloroquine, oral or parenteral gold, sulfasalazine, leflunomide, azathioprine, cyclosporine, mycophenolate mofetil), but these medications were not permitted within 4 weeks of the first study drug administration.

Patients could not have a history of latent or active tuberculosis (TB) prior to screening and were screened using the QuantiFERON-TB Gold test or tuberculin skin test (if the former was not approved in that country) within 6 months before the first study agent administration and chest radiograph within 3 months before the first study agent administration. In addition, patients were excluded if they had received, or were expected to receive, any live virus or bacterial vaccination within 3 months prior to the first administration of study agent, during the study, or within 6 months after the last administration of study agent.

Eligible patients were randomized (2:1) to receive IV infusions of golimumab 2 mg/kg at weeks 0, 4, and every 8 weeks thereafter or placebo at weeks 0, 4, and 12, with crossover to golimumab 2 mg/kg at weeks 24, 28, and every 8 weeks thereafter through week 100. Patients in the placebo plus MTX group with < 10% improvement in swollen and tender joint counts from baseline to week 16 entered early escape and received golimumab at weeks 16, 20, and every 8 weeks. All patients continued to receive a stable dose of MTX (15-25 mg/week).

### Statistical methods

This analysis included only patients who received ≥ 1 IV golimumab administration, and patients were grouped according to age: < 65 years or ≥ 65 years, < 70 years or ≥ 70 years, and < 75 years or ≥ 75 years. Efficacy was determined using the American College of Rheumatology (ACR) criteria. The proportion of patients who achieved ≥ 20%, 50%, or 70% improvement in the ACR criteria (ACR20/ACR50/ACR70 response) was determined for patients < 65 years or ≥ 65 years; nominal *p* values (chi-square test) were generated for comparisons between treatment groups in each age group separately without adjustment for multiplicity. Nonresponder imputation was used for patients who met the treatment failure or early escape criteria. For patients with missing data, last observation carried forward was used for ACR components. Physical function was evaluated using the Health Assessment Questionnaire-Disability Index (HAQ-DI) [19] and general health-related quality of life (HRQoL) and 36-item Short-Form Health Survey Physical and Mental Component Summary (SF-36 PCS/MCS) scores [20]. ACR response and change in HAQ-DI were determined for weeks 14, 24, 52, and 100; change in SF-36 PCS and MCS scores was determined for weeks 12, 24, 52, and 112. Efficacy analyses were not performed for the higher age cutoffs (70 year and 75 years) due to the small numbers of patients in these groups. Safety events through 2 years were summarized for patients < 65 years or ≥ 65 years, patients < 70 years or ≥ 70 years, and patients < 75 or ≥ 75 years.

## Results

### Baseline demographic and disease characteristics

The GO-FURTHER study was conducted at 92 sites in 13 countries (Argentina, Australia, Columbia, Hungary, Korea, Lithuania, Malaysia, Mexico, New Zealand, Poland, Russia, Ukraine, and the USA). Patients were randomized to receive placebo plus MTX (*n* = 197) or golimumab plus MTX (*n* = 395) at baseline. Demographic and disease characteristics were well-balanced between the treatment groups [18]. In this analysis, there were 515 patients aged < 65 years and 77 patients ≥ 65 years. Baseline demographics and disease characteristics for these patients are reported in Table 1. As expected, the mean ages differed between patients < 65 years and those ≥ 65 years. The proportions of patients receiving oral corticosteroids, NSAIDs, and DMARDs were lower among those aged ≥ 65 years. Patients ≥ 65 years had a longer mean RA disease duration and a slightly lower mean dose of oral corticosteroids at baseline compared with patients < 65 years. MTX use was categorized by time periods of (< 1 year, 1 to < 3 years, ≥ 3 years), and a greater proportion of younger patients seemed to be receiving MTX for longer than 3 years compared with patients ≥ 65 years. Other demographic and disease characteristics, including the ACR core assessments, were similar between these patient age groups.

**Table 1:** Baseline demographic and disease characteristics for patients < 65 years and ≥ 65 years

	Patients < 65 years			Patients ≥ 65 years		
	Placebo + MTX	Golimumab + MTX	Combined	Placebo + MTX	Golimumab + MTX	Combined
Patients, *n*	179	336	515	18	59	77
Age, years	49.5 ± 9.9	48.7 ± 10.7	49.0 ± 10.4	70.4 ± 3.4	70.1 ± 4.3	70.2 ± 4.1
Female	141 (78.8)	276 (82.1)	417 (81.0)	16 (88.9)	50 (84.7)	66 (85.7)
Race						
Caucasian	145 (81.0)	272 (81.2)	417 (81.1)	15 (83.3)	43 (72.9)	58 (75.3)
Asian	10 (5.6)	30 (9.0)	40 (7.8)	2 (11.1)	1 (1.7)	3 (3.9)
Black	0 (0.0)	0 (0.0)	0 (0.0)	0 (0.0)	1 (1.7)	1 (1.3)
Other	24 (13.4)	33 (9.8)	57 (11.1)	1 (5.6)	14 (23.7)	15 (19.5)
Weight, kg	71.7 ± 17.4	72.4 ± 16.2	72.2 ± 16.6	73.8 ± 15.3	66.3 ± 14.3	68.1 ± 14.7
BMI, kg/m^2^	26.9 ± 5.7	27.0 ± 5.6	27.0 ± 5.6	28.3 ± 5.5	25.8 ± 4.9	26.4 ± 5.1
RA disease duration	6.6 ± 6.3	6.5 ± 6.3	6.5 ± 6.3	10.8 ± 13.1	9.3 ± 9.8	9.7 ± 10.6
ACR core components						
Number of swollen joints (0–68)	14.7 ± 8.4	15.0 ± 8.5	14.9 ± 8.5	15.9 ± 9.8	14.6 ± 6.2	14.9 ± 7.2
Numbers of tender joints (0–68)	25.9 ± 14.3	26.6 ± 13.7	26.3 ± 13.9	25.8 ± 12.8	25.5 ± 15.2	25.6 ± 14.6
CRP, mg/dL	2.3 ± 1.9	2.8 ± 2.7	2.6 ± 2.5	1.5 ± 1.1	3.1 ± 3.7	2.7 ± 3.4
Physician’s global assessment (VAS, 0–10 cm)	6.3 ± 1.5	6.3 ± 1.6	6.3 ± 1.6	5.9 ± 2.0	6.0 ± 1.7	5.9 ± 1.7
Patient’s global assessment (VAS, 0–10 cm)	6.5 ± 1.9	6.5 ± 1.8	6.5 ± 1.9	6.2 ± 2.2	6.3 ± 1.8	6.3 ± 1.9
Patient’s assessment of pain (VAS, 0–10 cm)	6.5 ± 2.0	6.5 ± 1.9	6.5 ± 1.9	6.6 ± 2.0	6.5 ± 1.6	6.5 ± 1.7
HAQ-DI	1.60 ± 0.60	1.55 ± 0.66	1.56 ± 0.64	1.35 ± 0.72	1.61 ± 0.71	1.55 ± 0.72
Anti-CCP antibodies	165/177 (93.2)	307/335 (91.6)	472/512 (92.2)	16 (88.9)	55 (93.2)	71 (92.2)
Rheumatoid factor	164 (91.6)	309 (92.0)	473 (91.8)	17 (94.4)	56 (94.9)	73 (94.8)
SF-36 PCS	30.8 ± 7.2	31.0 ± 6.6	30.9 ± 6.8	31.7 ± 8.9	30.0 ± 7.8	30.4 ± 8.0
SF-36 MCS	38.3 ± 11.7	36.8 ± 11.1	37.3 ± 11.3	41.0 ± 10.4	38.9 ± 11.1	39.4 ± 10.9
Concomitant medications						
MTX dose at screening	16.7 ± 2.8	16.9 ± 2.9	16.8 ± 2.9	16.4 ± 2.9	16.3 ± 2.8	16.3 ± 2.8
Duration of MTX use						
< 1 year	44 (24.6)	82 (24.4)	126 (24.5)	4 (22.2)	20 (33.9)	24 (31.2)
1 to < 3 years	53 (29.6)	97 (28.9)	150 (29.1)	8 (44.4)	17 (28.8)	25 (32.5)
≥ 3 years	82 (45.8)	154 (45.8)	236 (45.8)	6 (33.3)	22 (37.3)	28 (36.4)
Unknown	0 (0.0)	3 (0.9)	3 (0.6)	0 (0.0)	0 (0.0)	0 (0.0)
Oral corticosteroids	121 (67.6)	221 (65.8)	342 (66.4)	13 (72.2)	30 (50.8)	43 (55.8)
Dose (prednisone or equivalent), mg/day	7.0 ± 2.5	7.1 ± 2.5	7.0 ± 2.5	6.9 ± 2.7	6.6 ± 2.7	6.7 ± 2.6
NSAIDs	145 (81.0)	280 (83.3)	425 (82.5)	11 (61.1)	43 (72.9)	54 (70.1)
Prior medications						
DMARDs*	83 (46.4)	182 (54.2)	265 (51.5)	9 (50.0)	24 (40.7)	33 (42.9)

Data presented as *n* (%) or mean ± standard deviation, unless otherwise noted

*ACR* American College of Rheumatology, *BMI* body mass index, *CCP* cyclic citrullinated peptide, *CRP* C-reactive protein, *DMARDs* disease-modifying anti-rheumatic drugs, *HAQ-DI* health assessment questionnaire-disability index, *MTX* methotrexate, *NSAIDs* nonsteroidal anti-inflammatory drugs, *RA* rheumatoid arthritis, *SF-36 PCS/MCS* 36-item Short Form Health Survey Physical/Mental Component Summary, *VAS* visual analog scale

*DMARDs other than MTX were discontinued ≥ 4 weeks prior to the first study agent administration

### Efficacy

**Fig. 1:**
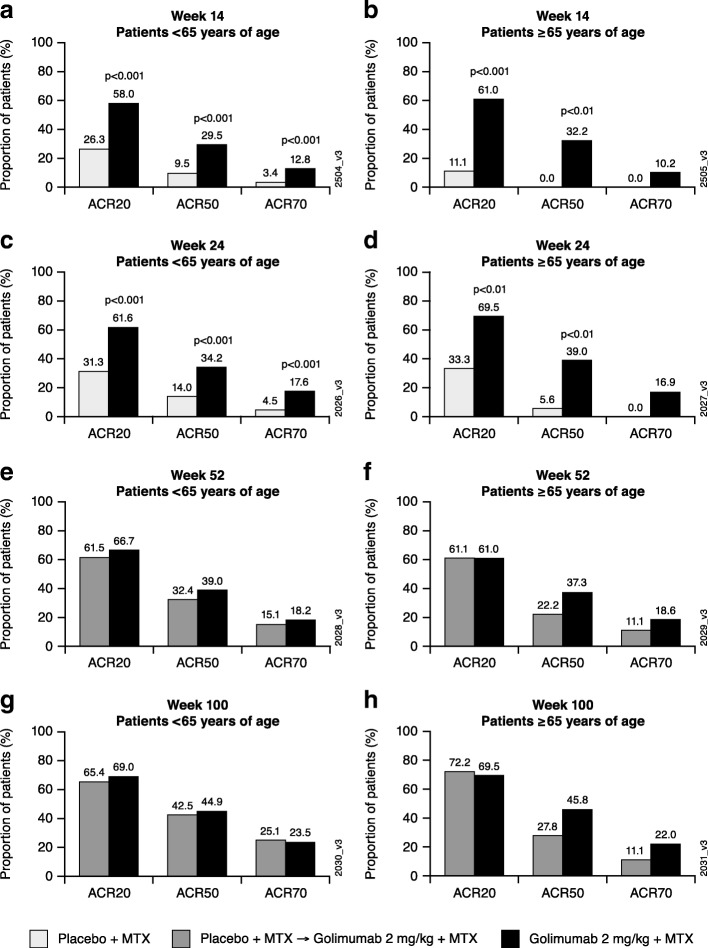
Proportions of patients < 65 years and ≥ 65 years achieving ACR20, ACR50, and ACR70 responses at weeks 14 (**a**, **b**), 24 (**c**, **d**), 52 (**e**, **f**), and 100 (**g**, **h**). Patients in the placebo group could receive golimumab at week 16 if they met the early escape criteria; all other patients in the placebo group crossed over to golimumab at week 24. Treatment group comparisons were not performed after week 24. *ACR20/50/70* ≥ 20%/50%/70% improvement in American College of Rheumatology criteria

At weeks 14 and 24, greater proportions of golimumab-treated patients achieved an ACR20 and ACR50 response compared with placebo among patients aged < 65 years and those ≥ 65 years. In addition, greater proportions of golimumab-treated patients achieved an ACR70 response compared with placebo in both age groups; however, the difference between treatment groups did not reach statistical significance among patients ≥ 65 years (Fig. 1). At weeks 52 and 100, when all patients had been receiving golimumab plus MTX since week 24, the proportions of patients achieving ACR20, ACR50, and ACR70 responses were similar for patients < 65 years and those ≥ 65 years within each treatment group (Fig. 1).

Mean improvements in HAQ-DI scores were also greater in the golimumab group compared with placebo in patients < 65 years and patients ≥ 65 years at weeks 14 and 24 (Table 2). Mean improvements in SF-36 PCS and MCS scores were greater in the golimumab-treated patients compared with placebo at weeks 12 and 24; however, differences between the treatment groups did not always reach significance among patients ≥ 65 years (Table 2). Among patients < 65 years, mean improvements in HAQ-DI and SF-36 PCS and MCS scores were sustained in the golimumab group through weeks 52 and 100/112, and improvements in the placebo crossover group approached those of the golimumab group at weeks 52 and 100/112. Among patients ≥ 65 years, improvements in HAQ-DI and SF-36 PCS and MCS scores were maintained through week 100/112 in the golimumab group. Patients who crossed over from placebo to golimumab also demonstrated improvements at weeks 52 and 100/112, although these improvements were smaller than those in the golimumab group; however, the small number of patients ≥ 65 years in the placebo crossover group limits the interpretation of these results.

**Table 2:** Physical function and health-related quality of life through week 100/112

	Patients < 65 years		Patients ≥ 65 years	
	Placebo + MTX ➔ Golimumab + MTX (*n* = 179)	Golimumab + MTX (*n* = 336)	Placebo + MTX ➔ Golimumab + MTX (*n* = 18)	Golimumab + MTX (*n* = 59)
Improvement from baseline in HAQ-DI				
Week 14	0.20 ± 0.57	0.50 ± 0.56***	0.10 ± 0.35	0.50 ± 0.65*
Week 24	0.22 ± 0.56	0.53 ± 0.62***	0.07 ± 0.43	0.53 ± 0.71**
Week 52	0.45 ± 0.60	0.53 ± 0.65	0.11 ± 0.41	0.40 ± 0.63
Week 100	0.49 ± 0.63	0.55 ± 0.67	0.29 ± 0.56	0.44 ± 0.60
Mean change from baseline in SF-36 PCS				
Week 12	3.3 ± 7.4	5.9 ± 7.5***	2.1 ± 7.3	6.0 ± 8.7
Week 24	4.0 ± 7.2	8.2 ± 8.2***	2.3 ± 8.6	8.9 ± 8.9**
Week 52	7.1 ± 8.0	8.2 ± 8.8	4.4 ± 8.7	7.4 ± 8.9
Week 112	7.2 ± 8.7	7.7 ± 9.0	5.7 ± 6.3	6.8 ± 10.1
Mean change from baseline in SF-36 MCS				
Week 12	1.6 ± 10.2	5.3 ± 10.0***	0.02 ± 5.80	2.9 ± 11.5
Week 24	1.2 ± 10.3	7.1 ± 10.2***	1.7 ± 7.6	6.0 ± 10.7
Week 52	4.1 ± 11.4	7.1 ± 11.0	2.0 ± 9.2	5.8 ± 12.2
Week 112	3.9 ± 11.5	6.0 ± 11.3	1.7 ± 9.2	4.4 ± 10.7

All data are presented as mean ± standard deviation

*HAQ-DI* health assessment questionnaire-disability index, *MTX* methotrexate, *SF-36 PCS/MCS* 36-item Short Form Health Survey Physical/Mental Component Summary

*p < 0.05, **p < 0.01, ***p < 0.001

### Safety

Through week 24, nine patients (2.2%) in the golimumab plus MTX group discontinued study treatment due to an AE. Of these, six patients (1.8%) were < 65 years at baseline, and three (5.1%) were ≥ 65 years of age. Through week 52 in the golimumab plus MTX group, 11 patients (3.3%) < 65 years and four patients (6.8%) ≥ 65 years discontinued study treatment due to AEs; through week 112, 23 patients (6.8%) < 65 years and 9 patients (15.3%) ≥ 65 years discontinued study treatment due to AEs. Overall, 86 (16.7%) patients < 65 years and 20 (26.0%) patients ≥ 65 years discontinued study agent through week 112. The two most common reasons for discontinuation were AEs (*n* = 44; 6.2% of patients < 65 years, 15.6% of patients ≥ 65 years) and withdrawal of consent (*n* = 31; 5.2% of patients < 65 years, 5.2% of patients ≥ 65 years). Among patients < 70 years, 6.5% discontinued study agent due to an AE, and 5.4% withdrew consent; among patients ≥ 70 years, 21.1% discontinued due to an AE, 2.6% withdrew consent, and 2.6% discontinued due to lack of efficacy. Among patients < 75 years, 6.9% discontinued study agent due to an AE, and 5.3% withdrew consent; among patients ≥ 75 years, 40.0% discontinued due to an AE, and all other discontinuations were for reasons other than AEs, withdrawal of consent or lack of efficacy.

Infections were the most common type of AE through week 52. The incidence of infections was similar between patients < 65 years (214/508, 42.1%) and those ≥ 65 years (30/76, 39.5%) and between patients < 70 years (227/547, 41.5%) and ≥ 70 years (17/37, 45.9%). Patients ≥ 75 years had a higher incidence of infections through week 52 than did patients < 75 years (5/10, 50.0%, and 239/574, 41.6%, respectively); however, the number of patients ≥ 75 years was small. Similar results were observed for the incidence of infections through week 112 (Table 3). Through week 112, upper respiratory infections were the most common infection in patients < 65 years (*n* = 67/508; 13.2%) and those ≥ 65 years (*n* = 9/76; 11.8%). The incidence of cellulitis was slightly higher among patients ≥ 65 years (*n* = 4/76, 5.3%) compared with those < 65 years (*n* = 10/508, 2.0%). Among patients ≥ 65 years, other infections included fungal urinary tract infection (*n* = 1) and septic arthritis (*n* = 2). There were four cases of herpes zoster; three occurred in patients < 65 years and one in a patient ≥ 70 years, and none were classified as serious or severe. Among patients who received golimumab plus MTX, 16/508 (3.1%) patients < 65 years and 7/76 (9.2%) ≥ 65 years had an infusion reaction through week 112. Skin reactions were the most common type of reaction among patients < 65 years (*n* = 5); vascular disorders (hypertension and flushing) were the most common reaction among patients ≥ 65 years (*n* = 3). Two patients in each age group experienced an infusion reaction of headache. None were considered serious or severe. No patient aged ≥ 65 years discontinued golimumab therapy due to an infusion reaction; one golimumab-treated patient, aged 39 years, discontinued due to a nonserious infusion reaction (mild skin reaction).

**Table 3:** Adverse events through week 52 and week 112 for patients who received at least one administration of golimumab

	Patients < 65 years	Patients ≥ 65 years	Patients < 70 years	Patients ≥ 70 years	Patients < 75 years	Patients ≥ 75 years
Patients, *n*	508	76	547	37	574	10
Through week 52						
Mean duration of follow-up, weeks	43.5	43.2	43.6	41.8	43.5	40.5
Mean number of golimumab infusions	5.9	5.8	5.9	5.6	5.9	5.4
Patients with ≥ 1 AE	355 (69.9)	52 (68.4)	379 (69.3)	28 (75.7)	397 (69.2)	10 (100.0)
Patients with infections	214 (42.1)	30 (39.5)	227 (41.5)	17 (45.9)	239 (41.6)	5 (50.0)
Patients with ≥ 1 SAE	45 (8.9)	9 (11.8)	49 (9.0)	5 (13.5)	50 (8.7)	4 (40.0)
Patients with serious infections	7 (1.4)	4 (5.3)	10 (1.8)	1 (2.7)	10 (1.7)	1 (10.0)
Through week 112						
Mean duration of follow-up, weeks	96.6	90.9	96.4	88.7	96.2	76.9
Mean number of golimumab infusions	12.0	11.3	12.0	10.9	12.0	9.4
Patients with ≥ 1 AE	417 (82.1)	61 (80.3)	445 (81.4)	33 (89.2)	468 (81.5)	10 (100.0)
Patients with infections	262 (51.6)	42 (55.3)	279 (51.0)	25 (67.6)	297 (51.7)	7 (70.0)
Patients with ≥ 1 SAE	90 (17.7)	19 (25.0)	97 (17.7)	12 (32.4)	102 (17.8)	7 (70.0)
Patients with serious infections	27 (5.3)	9 (11.8)	32 (5.9)	4 (10.8)	34 (5.9)	2 (20.0)

Data presented as *n* (%) unless otherwise noted

*AE* adverse event, *SAE* serious adverse event

The proportion of patients with at least one SAE through week 52 was numerically higher in patients ≥ 65 years (9/76, 11.8%) than in those aged < 65 years (45/508, 8.9%). Through week 52, the proportion of patients with at least one SAE was numerically higher for patients ≥ 70 years (5/37, 13.5%) compared with patients < 70 years (49/547, 9.0%) and for patients ≥ 75 years (4/10, 40.0%; cholecystitis and electrolyte imbalance [both in one patient], hemorrhoids, cerebral infarction, interstitial lung disease) compared with patients < 75 years (50/574, 8.7%). However, it should be noted that the numbers of patients ≥ 70 years and ≥ 75 years were relatively small. A similar trend was also observed for the proportions of patients with at least one AE through week 52.

Through week 112, 19 (25.0%) patients ≥ 65 years had an SAE, including cellulitis and bacterial arthritis. Among patients ≥ 75 years (*n* = 10), 7 (70.0%) had an SAE, including intervertebral discitis and diverticulosis. Ninety patients (17.7%) < 65 years had an SAE through week 112, including one patient with acute pancreatitis. Other SAEs through week 112 included fractures in eight golimumab plus MTX-treated patients: five who were < 65 years, one who was ≥ 65 and < 70 years (femoral neck fracture), one who was ≥ 70 and < 75 years (upper limb fracture), and one who was ≥ 75 years (spinal compression fracture; same patient with the intervertebral discitis). Additionally, among patients treated with golimumab plus MTX, six malignancies were reported through week 112; all occurred in patients < 65 years (breast cancer, cervical carcinoma stage 0, basal cell carcinoma (*n* = 2), Bowen’s disease, and chronic lymphocytic leukemia).

In the overall GO-FURTHER population, 36 golimumab plus MTX-treated patients (6.2%) experienced a serious infection through week 112 [7]. In this post hoc analysis by age, there was a higher incidence of serious infections among patients ≥ 65 years, ≥ 70 years, and ≥ 75 years when compared with patients < 65 years, < 70 years, and < 75 years, respectively, through weeks 52 and 112; however, the numbers of patients ≥ 70 years and ≥ 75 years were small. Serious infections included sepsis (*n* = 2), septic arthritis (*n* = 1), and cellulitis (*n* = 1) among patients ≥ 65 years and rectal abscess (*n* = 1), sepsis (*n* = 1), and urosepsis (*n* = 1) among patients < 65 years. Two serious opportunistic infections were reported: infective spondylitis (*Candida albicans*) in a patient ≥ 75 years and a serious infection of cryptococcal pneumonia in a patient ≥ 65 years from South Korea. Three cases of active tuberculosis were reported through week 112, all in patients younger than 65 years. Through week 112, seven patients experienced an SAE of pneumonia; all were younger than 65 years, with the exception of the patient with cryptococcal pneumonia. There was no predominant type of serious infection, including opportunistic infections, among patients ≥ 65 years. Among patients who did not use corticosteroids at baseline, the incidence of serious infections through week 112 was similar for patients < 65 years and ≥ 65 years (13/170, 7.6%, and 3/33, 9.1%, respectively), while among patients who received oral corticosteroids at baseline, the incidence of serious infections was numerically higher for patients ≥ 65 years (6/43, 14.0%) compared with those < 65 years (14/338, 4.1%).

A total of five deaths occurred through 2 years in patients who received golimumab plus MTX [7, 18]: four occurred in patients who were younger than 65 years (myocardial infarction complicated by presumed pneumonia, abdominal tuberculosis, septic shock, and unknown causes) and one occurred in a patient older than 65 years (*Clostridium difficile*). One death occurred in a patient receiving placebo plus MTX (< 65 years; presumed stroke secondary to hypertensive crisis) as previously reported [18].

## Discussion

Maintenance of efficacy through 2 years of treatment with IV golimumab 2 mg/kg plus MTX in the GO-FURTHER trial of biologic-naïve patients with RA has been previously demonstrated [7, 18]. In the current analysis, greater proportions of golimumab plus MTX-treated patients achieved an ACR20 response at weeks 14 and 24 compared with placebo plus MTX both in patients < 65 years and those ≥ 65 years. A similar treatment effect at week 24 was observed in both age groups. At weeks 52 and 100, ACR response rates remained similar for both age groups. Likewise, improvements in physical function and HRQoL were greater among golimumab-treated patients compared with placebo in both age groups through week 24. Among patients ≥ 65 years, those in the placebo group generally had smaller improvements in physical function and HRQoL even after crossing over to golimumab. Of note, the number of patients in this group was small (*n* = 18), mean age was 70 years, and these patients had a higher mean disease duration compared with patients < 65 years, which may have affected these outcome measures.

Through week 112, the proportions of golimumab plus MTX-treated patients with at least one AE were similar for patients < 65 years and those ≥ 65 years; similar results were observed when using the 70 years and 75 years age cutoffs. Infections were the most common type of AE, which is consistent with the known safety profile of golimumab. The proportions of patients with an SAE were numerically higher in the patients ≥ 65 years, ≥ 70 years, and ≥ 75 years compared with those < 65 years, < 70 years, and < 75 years, respectively. Similar trends were also seen for serious infections. Few patients experienced infusion reactions with golimumab (< 65 years: 16/508, 3.1%; ≥ 65 years: 7/76, 9.2%). None of the infusion reactions were considered serious or severe, and there were no discontinuations of study treatment due to an infusion reaction among patients ≥ 65 years. However, these comparisons should be interpreted with caution due to the relatively small population sizes in the older age groups.

The use of anti-TNF therapy has been associated with an increased risk of infection [21] and an increased risk of some malignancies compared with the general population [22]. Evaluating safety, particularly infections, in older patients is complicated by the changes in the immune system that occur with increasing age [23]. Immunosenescence is known to affect older patients, and the presence of a chronic condition further increases the risk of infection in these patients [24]. In a retrospective study of patients with RA who were receiving anti-TNF therapy, discontinuation of treatment due to an AE was more common among patients ≥ 65 years of age than in younger patients [25]. A higher incidence of serious infections has been seen in elderly patients with RA treated with anti-TNF agents (infliximab, adalimumab, etanercept) [26] and Janus kinase inhibitors (tofacitinib [27] or baracitnib [28]).

Among patients who were using concomitant corticosteroids at baseline, the incidence of serious infections was numerically higher for patients ≥ 65 years than for patients < 65 years. No apparent difference between age groups was observed for patients who were not receiving corticosteroids at baseline. This is consistent with an increased risk of infections, such as viral infections, tuberculosis, sepsis, and bacterial pneumonia, observed in patients receiving oral corticosteroids [29]. This offers a clear message to clinicians to strive to reduce and/or eliminate concomitant use of corticosteroids in elderly patients. This post hoc analysis is limited by the relatively small patient numbers in the older age groups. In addition, these results may have been confounded by the effect of immunosenescence in these patients.

## Conclusions

The results from this exploratory analysis suggest that clinical efficacy of IV golimumab 2 mg/kg plus MTX is similar between patients ≥ 65 years and those < 65 years. We observed a slightly higher numeric incidence of serious infections for patients ≥ 65 years, ≥70 years, and ≥ 75 years compared with the younger age groups. In general, the types of AEs observed in the older age groups were consistent with the known safety profile of golimumab. However, it should be noted that the results of this post hoc analysis are based on small patient numbers, particularly in the age groups ≥ 70 years and ≥ 75 years. Additionally, the increased risk of infection with age due to immunosenescence complicates the interpretation of these results in older patients. Although this analysis did not demonstrate a stronger signal for serious infections, these results do advise cautious patient selection for risk and monitoring for serious infection for older patients receiving golimumab. This is especially true with the use of concomitant corticosteroids, which in this study, was associated with a higher incidence of serious infections, consistent with findings from other studies that examined older age patients.

## Data Availability

The data sharing policy of Janssen Pharmaceutical Companies of Johnson & Johnson is available at https://www.janssen.com/clinical-trials/transparency. As noted on this site, requests for access to the study data can be submitted through Yale Open Data Access (YODA) Project site at http://yoda.yale.edu.

## Abbreviations

ACR: American College of Rheumatology
AE: Adverse event
AS: Ankylosing spondylitis
DMARDs: Disease-modifying anti-rheumatic drugs
HAQ-DI: Health Assessment Questionnaire-Disability Index
HRQoL: Health-related quality of life
IV: Intravenous
MTX: Methotrexate
NSAIDs: Nonsteroidal anti-inflammatory drugs
PsA: Psoriatic arthritis
RA: Rheumatoid arthritis
SAE: Serious adverse event
SC: Subcutaneous
SF-36 PCS/MCS: 36-item Short-Form Health Survey Physical and Mental Component Summary
TB: Tuberculosis
TNF: Tumor necrosis factor
